# A case report on the management of congenital urethroperineal fistula in an adolescent, a rare congenital anomaly

**DOI:** 10.1016/j.ijscr.2025.111043

**Published:** 2025-02-11

**Authors:** Samuel Fekadu Shiferaw, Mezgeb Gedefe Molla, Yoseph Abebe Feye

**Affiliations:** a Addis Ababa University, CHS, School of Medicine, Department of Surgery, Urology Unit, Addis Ababa, Ethiopia; b Menelik II Comprehensive Specialized Hospital, Department of Surgery, Addis Ababa, Ethiopia

**Keywords:** CUPF, Ethiopia, Duplicate urethra, Congenital fistula, Rare case

## Abstract

**Introduction:**

Congenital posterior urethra perineal fistula (CUPF) is a rare congenital GU anomaly with abnormal epithelium-lined communication between the posterior urethra and perineum. Some consider it to be a variant of Y-type urethral duplicate with ventral hypoplastic urethra and have suggested its adoption to the Eiffmans classification.

**Case presentation:**

An 18-year-old male patient presented with urine dribbling from the perineum since childhood. He has no history of urinary tract infection and surgical procedure and instrumentation. There was normal external genitalia with meatus positioned at the tip of the glans; there was a small opening on the posterior scrotal wall near the perineum that admits a 6fr NG tube. VCUG showed a fistula from the prostatic urethra to the perineum. He was managed with near-total excision of the fistula tract, with no recurrence on a 3-month follow-up.

**Discussion:**

Patients with this rare condition present with dribbling from perineum during voiding, underwear wetting and recurrent UTI. Voiding CUG is a diagnostic procedure of choice for patients with CUPF. MRI can be used if there is interest in detail anatomy of the fistula tract and its relationship to the surrounding structures and is also helpful if other modalities show a non-conclusive result. In managing this patient, differentiating the dominant and functional urethra is of clinical significance. Complete or partial excision, electrofulgration of the fistula tract are options.

**Conclusion:**

Voiding CUG is a diagnostic procedure of choice for patients with CUPF and partial excision of the fistula tract is an option of management with satisfactory result.

## Highlights

• CUPF is a rare congenital anomaly characterized by a fistulous communication between the prostatic urethra and perineum.
• Less than 40 cases have been reported in the English Literature up-to-date.
• Voiding CUG will diagnose most cases.
• Partial excision of the fistula tract is an alternative option of management.

## 1 Introduction

Congenital urethroperineal fistula (CUPF) is a rare congenital GU anomaly where there is an abnormal epithelium-lined fistulous communication between the prostatic urethra, originating just below the bladder neck from or near the verumontanum, and perineum [[1], [2], [3]]. It resembles the Type IIA2, Y-type duplication of the Eiffman classification but with a hypoplastic ventral and functional dorsal urethra [4]. Diagnosis is usually made by voiding cystourethrography (VCUG) or retrograde urethrography (RUG) with fistulography. Cystourethroscopy with dye injection through the fistula adds to the confirmation of diagnosis. MRI can be used to evaluate the anatomy of the tract and its relationship to the surrounding structures or if other modalities show a nonconclusive result [2,[5], [6], [7]]. Successful endoscopic and surgical management options have been reported, but differentiating the functional urethra is the most important factor in determining the management [[2], [3], [4], [5],^7^,^8^].

This report discusses the first reported CUPF case in Ethiopia in a young boy in his late adolescence. He was managed at a public hospital with a partial fistula tract excision with no recurrence on his 3rd month post-operative follow-up. This report is prepared according to the SCARE checklist criteria [9].

## 2 Presentation of case

This is an 18-year-old male who presented with a complaint of dribbling of urine via the perineum while urinating since his early childhood. He voids normally through the meatus at the tip of his glans. He has no known comorbid conditions and no history of urethral instrumentation, perineal trauma, previous surgery, or urinary tract infection treatment.

**Fig. 1:**
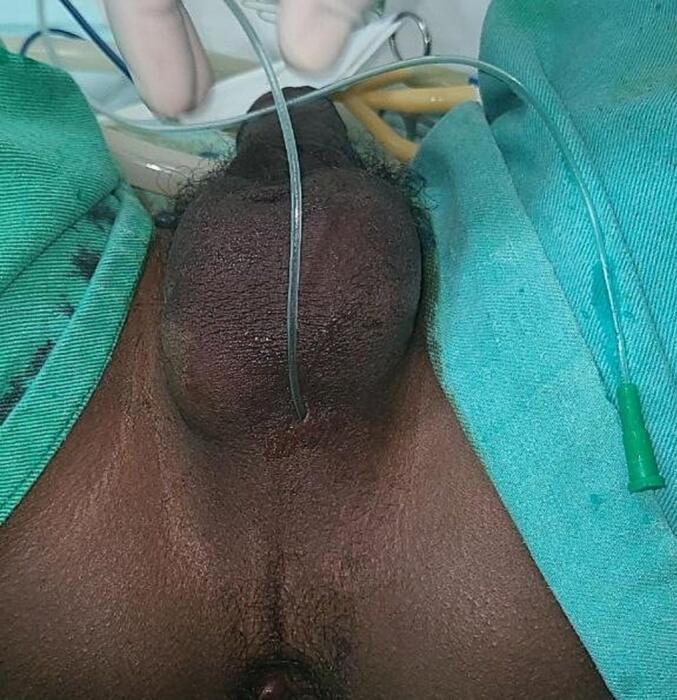
A 6fr NG tube inserted through the fistula tract.

At presentation, his vital signs were stable, and all physical examinations were unremarkable. He has a normal-sized meatus at the tip of his glans, and his penis is age-appropriate. There is a small midline opening at the junction of the posterior scrotal wall and perineum that permits a 6 Fr nasogastric feeding tube to pass through (Fig. 1).

**Fig. 2:**
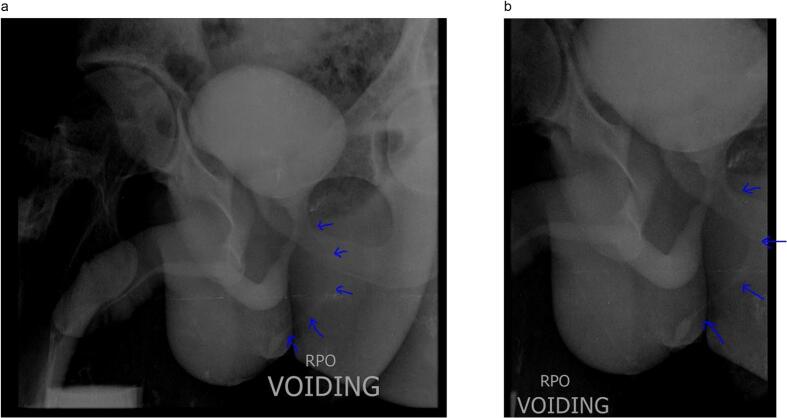
a: Voiding phase of a CUG showing fistula tract (blue arrow). b: Voiding phase of a CUG showing fistula tract (blue arrow). (For interpretation of the references to colour in this figure legend, the reader is referred to the web version of this article.)

A complete blood count, renal function test, urine analysis, and abdominal ultrasound were performed on him; all of the results were within normal limits and showed no abnormalities. Following a cystourethroscopy, the urethra was found to be normal, with the external urinary sphincter and verumontanum intact with no visible internal fistula orifice. A CUG was performed, and during the voiding phase, it revealed a fistulous tract that extends from the prostatic urethra close to the neck of the bladder to the perineum (Fig. 2a, b).

**Fig. 3:**
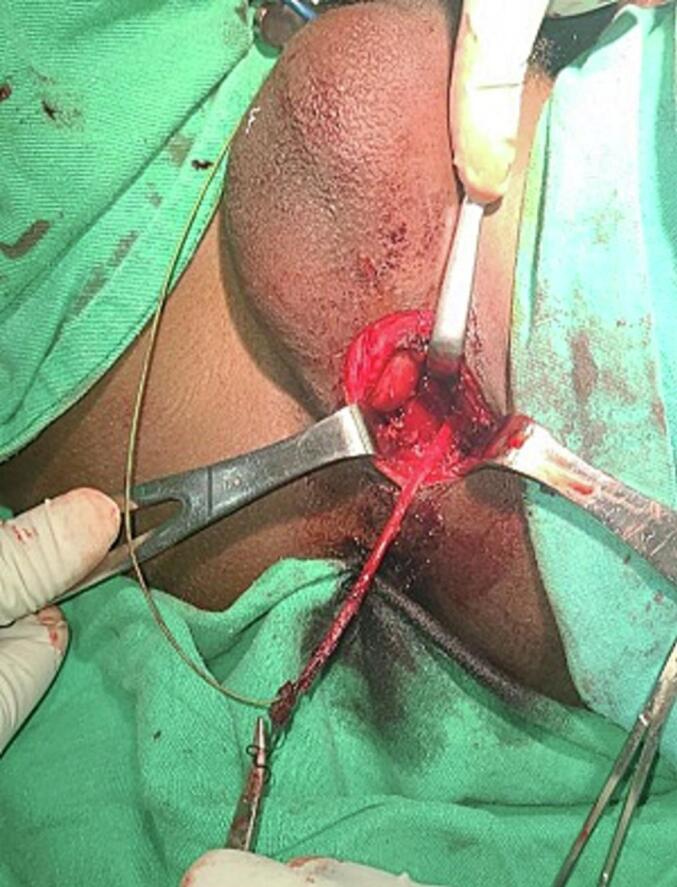
Fistula tract dissected as high as possible, resected and ligated proximally.

**Fig. 4:**
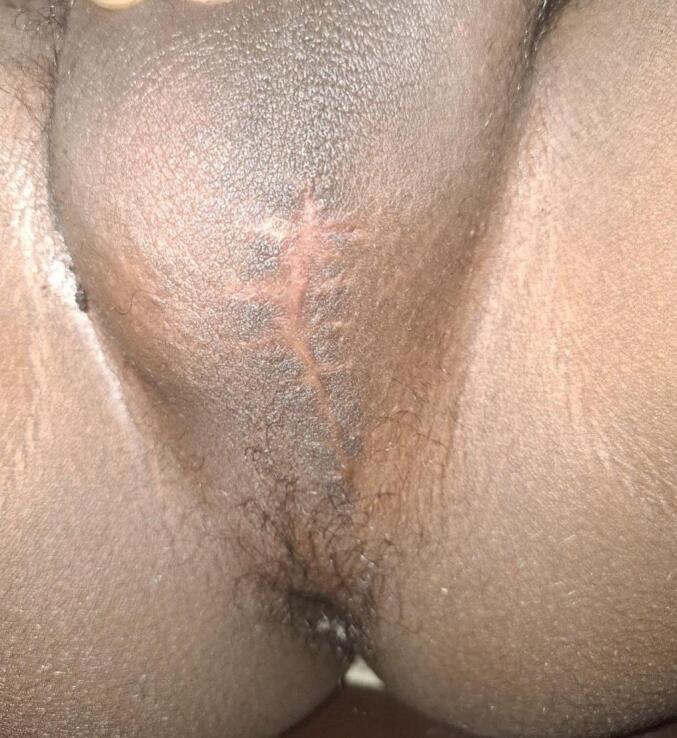
Postoperative picture of the premium showing a healed wound.

The Patient was diagnosed with CUPF with functional dorsal urethra and stenotic ventral fistula. A partial excision of the fistula was performed under spinal anesthesia. The patient was positioned in the lithotomy position, and a 6fr nasogastric feeding tube was used to cannulate the fistula tract. This tube was transfixed at the orifice of the fistula to serve as a traction guide during the dissection. A 4 cm midline vertical incision was made encircling the fistulous tract, and the tract was dissected from the surrounding tissue using the nasogastric tube for guidance and traction up to the pubic symphysis. The dissected fistula tract was ligated proximally with a 2-0 Vicryl suture and subsequently removed (Fig. 3). The fistula tract had a curvilinear path, arising from the midline on the median raphe, continuing posteriorly to the left side, lateral to the bulbospongiosus, and then returning anteriorly to the midline. There was no intraoperative or postoperative complication. The patient was discharged on the first postoperative day with a transurethral catheter, prescribed oral ciprofloxacin for 7 days, and advised to take sitz baths three times a day for 5 days. Two weeks after the procedure, the catheter was removed, and the patient experienced good voiding with a healed perineal wound (Fig. 4). A follow-up after 3 months revealed no recurrence of urine dribbling from the perineum and no urinary complaints from the patient. The pathological specimen showed a 7.5 cm tubular structure with a squamous epithelial lining and a surrounding smooth muscle layer.

## 3 Discussion

CUPF is a rare congenital GU anomaly where there is an abnormal epithelium-lined fistulous communication between the prostatic urethra, originating just below the bladder neck from or near the verumontanum, and perineum [[1], [2], [3]]. Reports of this condition have appeared across various geographic regions and age groups, but fewer than 40 cases have been documented to date [4,5,10]. Based on our comprehensive literature review, we believe our case is the first of its kind reported in this country. While CUPF has been associated with other anomalies such as anorectal malformations, hypospadias and penile curvature the patient in this case has no associated gastrointestinal or genitourinary abnormalities [7]. There are multiple proposed theories for the development of the different types of duplications [11]. There are several theories regarding the development of various types of duplications. One theory posits that CUPF may arise from a focal or temporary defect in the urethral plate, which impedes the fusion of the urethral folds. Another theory highlights the possibility of pressure atrophy resulting from the heel of the fetal foot [8].

CUPF is similar to the Type IIA2 Y-type duplication as described in the Eiffman classification. However, CUPF is characterized by a hypoplastic ventral urethra and a functional dorsal urethra. Due to their close relationship, some authors have suggested that CUPF should be included in Eiffman's classification system for urethral duplications as Y-type duplication with a hypoplastic ventral urethra [5]. A systematic review conducted by Horea et al. found that Type IIA2 Y-duplication is the most common type of urethral duplication, with a prevalence of 35 % (91 out of 250 cases). Of these, only six patients had a dominant and functional dorsal urethra paired with a stenotic ventral urethra, which aligns with the definition of CUPF found in the literature [1]. Although distinguishing CUPF from duplicate urethra can be challenging, identifying the functional urethra is critical for treatment. Typically, the functional urethra has a wide caliber for effective bladder drainage, a proper sphincter mechanism, and a normal verumontanum [1,2,5,8,11].

Our patient presented with intermittent perineal discharge during urination since childhood, similar to most cases. Dysuria with urinary tract infection and underwear wetting are also documented in other patients. Usually the diagnosis of CUPF is made later than in patients with urethral duplication which may be due to the fact that urine mainly exits through the meatus at the glans tip [2,5,10].

Diagnosis is typically confirmed with voiding cystourethrogram (VCUG). While retrograde urethrography (RUG) generally does not reveal the fistula tract, it can be paired with fistulography to demonstrate the characteristic Y configuration [2,5]. MRI is useful for mapping the anatomical course of the fistulous tract and assessing its relationship with surrounding pelvic and perineal tissues and vessels. It can also assist in diagnosis when other tests are inconclusive [2,7,10]. Cystourethroscopy may reveal the internal fistula opening; if not, dye instillation or cannulation with small tubes can help identify it [[5], [6], [7]]. Evaluating the upper urinary tract is advisable, as this condition may co-occur with renal, ureteral, or bladder anomalies [2,5]. In this instance, RUG and cystourethroscopy were normal; however, voiding CUG indicated an abnormal tract from the prostatic urethra to the perineal skin. Cystourethroscopy combined with dye injection through the fistula's external opening could aid in locating the internal orifice for potential endoscopic treatment.

Patients have been managed with various methods, including electrical fulguration, complete or partial tract excision, and closure of the fistula tract at its internal urethral orifice using bulking agent injection. Fulguration of the internal opening by electrode resulted in one recurrence, but fulguration of the entire tract was successful and showed no recurrence, along with a short hospital stay and low cost [10]. Partial fistula tract excision while preserving a small part near the prostatic urethral is helps to prevent urethral sphincter injury, prostatic urethra injury, and impotence. While complete excision of the tract is beneficial to prevent future malignancy from chronic irritation, dissection near the proximal tract is difficult due to limited exposure and fear of urethral sphincter injury, impotence and Prostatic urethra injury. Tract excision (Partial or complete) has been shown to be most effective with no recurrence [[2], [3], [4], [5],^7^]. Partial excision and proximal tract fulguration is also a viable option, given the challenges of dissection near the Proximal tract [3]. Endoscopic injection of bulking agent has been used in one patient with no recurrence during follow-up [8]. After consideration of the available alternative options and the resources at our disposal, our patient was managed with partial excision of the fistula tract. Notably, there has been no evidence of recurrence at the 3-month follow-up. The successful management of this case contributes to the existing body of evidence supporting partial excision as a viable and safe alternative, particularly in instances where complete removal of the tract is not feasible.

## 4 Conclusion

Voiding CUG is the preferred diagnostic procedure for patients with CUPF. MRI serves as a valuable tool in evaluating the anatomy of the fistula tract and clarifying its relationships with adjacent structures, particularly when other diagnostic methods yield inconclusive results. It is essential to distinguish between the dominant and functional urethra in the management of CUPF. Moreover, partial fistula excision is a viable and safe alternative, particularly in instances where complete removal of the tract is not feasible.

## Author contribution

1. Samuel Fekadu Shiferaw, MD, MPH, Urology resident: Conceived, wrote and submitted the case report. Operated on the patient and followed him postoperatively.
2. Mezgeb Gedefe, MD, Urologist: Contributed to the writing and reviewed the case report. Operated on the patient and followed him postoperatively.
3. Yoseph Abebe Feye MD, Urology resident: Reviewed the case report. Operated on the patient and followed him postoperatively.

## Consent

Written informed consent was obtained from the patient for publication of the case report and accompanying the images.

## Ethical approval

Ethical approval was provided by the authors' institution.

Ethical review committee of the Department of Surgery, College of Health Sciences, Addis Ababa University.

## Guarantor

Dr. Mezgeb Gedefe.

## Research registration number

Not applicable.

## Funding

There is no source of funding found for this research paper.

## Conflict of interest statement

N/A.
